# Melanopsin photoreception contributes to human visual detection, temporal and colour processing

**DOI:** 10.1038/s41598-018-22197-w

**Published:** 2018-03-01

**Authors:** Andrew J. Zele, Beatrix Feigl, Prakash Adhikari, Michelle L. Maynard, Dingcai Cao

**Affiliations:** 1 Institute of Health and Biomedical Innovation, Queensland University of Technology (QUT), Brisbane, Australia; 2 School of Optometry and Vision Science, Queensland University of Technology (QUT), Brisbane, Australia; 3 School of Biomedical Sciences, Queensland University of Technology (QUT), Brisbane, Australia; 4 Queensland Eye Institute, Brisbane, Australia; 5 Department of Ophthalmology and Visual Sciences, University of Illinois at Chicago, Chicago, USA

## Abstract

The visual consequences of melanopsin photoreception in humans are not well understood. Here we studied melanopsin photoreception using a technique of photoreceptor silent substitution with five calibrated spectral lights after minimising the effects of individual differences in optical pre-receptoral filtering and desensitising penumbral cones in the shadow of retinal blood vessels. We demonstrate that putative melanopsin-mediated image-forming vision corresponds to an opponent S-OFF L + M-ON response property, with an average temporal resolution up to approximately 5 Hz, and >10x higher thresholds than red-green colour vision. With a capacity for signalling colour and integrating slowly changing lights, melanopsin-expressing intrinsically photosensitive retinal ganglion cells maybe the fifth photoreceptor type for peripheral vision.

## Introduction

Melanopsin photoreception in intrinsically photosensitive retinal ganglion cells (ipRGC) has fundamental roles in light dependent, non-imaging forming (i.e. non-visual) functions such as circadian photoentrainment and pupil light responses^1–4^. Anatomical studies in macaque show that ipRGCs project to the lateral geniculate nucleus (LGN) of the thalamus^3^ in the image-forming (visual) pathways, however it is uncertain if the S-OFF/L + M-ON colour opponent response property of ipRGCs can subserve vision. Functional magnetic resonance imaging studies in humans to high contrast melanopsin directed stimuli elicit a response in the visual cortex (area V1) which are associated with a brightening of visual percepts and distinct from the perceptual response to cone luminance directed stimuli^5^. Brightness discrimination experiments show that cone metamers with higher melanopsin excitation are reliably judged as brighter than those with lower melanopsin excitation^6^, with brightness estimations resulting from a combined contribution from cone and melanopsin signalling^7^. The detection threshold for light stimuli modulated along a cone-silent direction attributed to the activation of melanopsin is also higher than thresholds for stimuli modulating the L-, M- or S-cone directions^8^. Temporal contrast responses to melanopsin directed stimuli under conditions silencing the activation of the rods and three cone types is low pass, with a high frequency cut-off at ~20 Hz^9^, yet this high frequency response could imply the intrusion of non-melanopsin photoreceptor absorptions, potentially via penumbral cones^8^ which become relevant at frequencies beyond ~4 Hz^10^.

To measure the perceptual correlate of melanopsin signalling in humans, the behavioural effects of melanopsin activation must be separated from visually detectable L- and M-cone signals arising through inadvertent stimulation of penumbral cones in the shadow of retinal blood vessels^10^. In this study we separate melanopsin signalling from penumbral cones using a technique of photoreceptor silent substitution with five calibrated spectral light distributions^9^ and minimise the effect of individual differences in pre-receptoral filtering due to optical components in the eye^9^. By doing so, penumbral cones can be desensitised with temporal white noise that modulates the L-cone, M-cone, S-cone and rod photoreceptor excitations (without changing the melanopsin photoreceptor excitation) to provide a direct measure of melanopsin photoreception in humans. With stimulus conditions designed to modulate the melanopsin response, we estimate the visual detection threshold for melanopsin, its temporal contrast response and characterise its purported colour opponent response property.

## Methods

### Participants

The Human Research Ethics Committee at Queensland University of Technology (QUT) approved all experimental procedures. Participants provided signed informed consent prior to the experiments. All participants were experienced psychophysical observers with trichromatic colour vision and no ophthalmic disease (n = 4; 22–41 years, 2 males, 2 females); two Authors (PA, MLM) and two participants naïve to the purpose of the study. Experiments were conducted in accordance with the ethics approval. Participants dark adapted for 10 min prior to all photopic measurements.

### Independently controlling the retinal photoreceptor excitations with a 5-primary optical photostimulator

**Figure 1:**
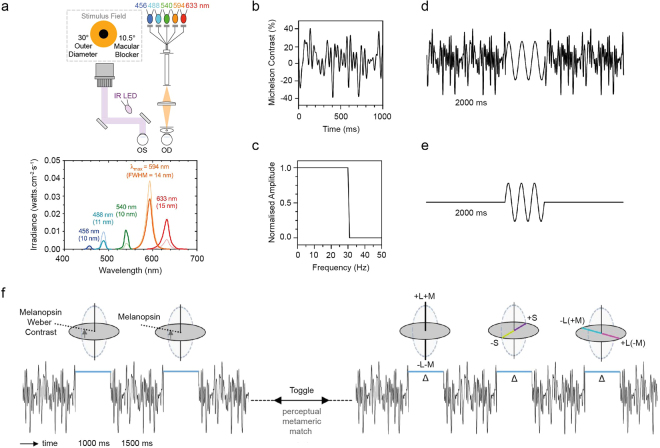
Optical photostimulator and test conditions. (**a**) Optical generation of 5 spectrally controlled primary lights for modulating the excitation of melanopsin, rhodopsin and the three cone opsins in the peripheral retina of the human eye (OD); consensual pupil light responses (OS) are recorded under infra-red (IR) illumination. The relative spectral outputs of the 5 primary lights (peak wavelength and full width at half maximum, FWHM) are shown for a 2000 Troland, orange appearing adapting field (thick lines), and a 17% Weber contrast increment in melanopsin excitation with no change in the excitation of the rods and cones (thin lines); see Supplementary Table S1. (**b**) Temporal white noise (TWN) modulated the S-cone, M-cone, L-cone and Rod photoreceptor excitations (40% Michelson contrast; 0–30 Hz noise) without changing the melanopsin photoreceptor excitation. (**c**) Temporal white noise has a constant power spectral density up to the 30 Hz cut-off. (**d**) Test sequence for the temporal contrast sensitivity paradigm with penumbral cone silencing temporal white noise. The sinusoidal flicker stimuli are melanopsin, S-cone or cone (L + M) luminance isolating and the frequency and contrast are variable (unmodulated photoreceptor classes are silenced). Visual detection thresholds were also measured with a 1000 ms incremental pulse stimulus. (**e**) Test sequence for the temporal contrast sensitivity paradigm without noise. Sinusoidal stimulus parameters are independently variable. (**f**) The colour appearance of melanopsin directed signalling was quantified with a psychophysical temporal colour matching technique in terms of cone inputs to visual pathways; equiluminant 3D colour spaces show the cone modulations along the three dimensions: +L + M and −L −M; +S and −S; +L − M and −L + M. Three adapting field colours (white, yellowish-pink and orange) were chosen to vary the saturation level and the relative L-, M- and S-cone photoreceptor excitations. Penumbral cone silencing temporal white noise was present between the melanopsin stimulus and variable cone increments.

Stimuli were generated using a custom-built 5-primary photostimulator^9^ (Fig. 1a) to independently control the excitations of melanopsin, rhodopsin and the three cone opsins^11–13^ using the principle of silent substitution^12,14^. The chromaticities of the stimuli were specified in a relative cone-Troland space which plots *l* = L/(L + M) versus *s* = S/(L + M) with a normalisation of *l* = 0.667 and *s* = 1.0 for an equal energy spectrum (EES light); melanopsin and rod excitations were both normalised to 1 at 1 photopic Troland (Td)^15^. Three adapting stimulus field chromaticities were chosen to vary the relative L-, M- and S-cone photoreceptor excitations at 2000 photopic Td; a white appearing field metameric to an EES light, a yellowish-pink appearing field (*l* = 0.750, *s* = 0.60, rod = 0.575 and melanopsin = 0.550) and an orange appearing field (*l* = 0.752, *s* = 0.105, rod = 0.319 and melanopsin = 0.235). Note that the baseline melanopsin excitation needed to decrease with increasing saturation due to instrument gamut limitations. Saturation was defined in terms of excitation purity, with a larger displacement from the white point (EES) towards the spectrum locus for the orange field (84.6%) than the yellowish-pink field (48.1%).

The primaries were generated using light-emitting diodes (LEDs) combined with narrow band interference filters^9^ yielding dominant wavelengths of 633 nm (red), 594 nm (amber), 540 nm (green), 488 nm (cyan) and 456 nm (blue). The stimulus field, a 30° outer diameter annulus with a central 10.5° macular blocker, was imaged in the plane of a 2 mm artificial pupil in Maxwellian view. Experiments were conducted with natural pupils. A pinhole through the centre of the macular block was used for fixation. The radiances of the primaries were controlled by an Arduino based stimulation system with an LED driver (TLC5940) and microcontroller (Arduino Uno SMD R3, Model A000073) with custom engineered software driven by an Apple MacPro QuadCore Intel computer. The pulse width modulation frequency generates stimuli up to ~488 Hz at 12-bit resolution per primary light^9^, well beyond the critical flicker fusion frequency of human vision and of cells in retinogeniculate visual pathways^16^. A physical light calibration measured the spectral output of the narrow-band primaries at 1 nm intervals using an EPP2000C-50um Slit UV-VIS Spectrometer (StellarNet, Tampa, FL, USA) (Fig. 1a). The linearisation coefficients for each primary were generated based on the luminance output of each LED and interference filter combination measured using an ILT1700 Research Radiometer (International Light Technologies, Inc., Peabody, MA, USA) as a function of the duty cycle of the LED driver^9^.

We compensated for individual differences in pre-receptoral filtering between the observer and the CIE 1964 10° standard observer by completing an individual observer calibration procedure. This calibration assumes that the photoreceptor spectral sensitivities of the observer at the primary wavelengths are approximately linear transforms of the standard observer colour matching functions, as previously demonstrated^17^. Heterochromatic Flicker Photometric settings at 15 Hz were obtained for primary light combinations and the sensitivity difference between the individual observer and the 10° standard observer was estimated by comparing the relative radiances of the settings for each primary light combination with the theoretical values required by the 10° standard observer. Stimulus contrasts were then calculated for participants using their individual spectral calibration data. Supplementary measurements confirmed the observer calibration and photoreceptor isolation. In one measure, a 500 ms, 18% Weber contrast rod pulse (no change in the melanopsin or cone excitations) at a 5 Troland mesopic adaptation level was invisible after photopigment bleach and highly conspicuous after dark adaptation. In another control measure, the cone excitations perceptually matching a 500 ms, 18% contrast rod increment pedestal (with no change in melanopsin excitation) at a 5 Troland adaptation level are equivalent to a decrease in L/[L + M], increase in S/[L + M] and an increase in [L + M]^18^. In addition, a 500 ms rod increment stimulus presented at the maximum achievable contrast (18.5%) was invisible on the 5000 photopic Troland background.

### Desensitising penumbral cones with temporal white noise

The absorption of light by the retinal vasculature means that open-field and penumbral cones require different spectral light distributions for photoreceptor isolation. To determine the penumbral L-, M-, and S-cone contrasts for the open-field photoreceptor isolation used in our experiments we estimated the effect of haemoglobin absorption^19^ on the primary lights following previous estimates^8,10^; oxyhaemoglobin (HbO_2_) and deoxyhaemoglobin (Hb) absorbance coefficients (*aHbO*_2_ and *aHb*) were defined as 5*[{2.303 * molar extinction coefficient * 0.*002326*)}/*1000*], where 5 µm is the average central retinal capillary diameter within 3.5 mm eccentricity (~23.4° field of view)^20^ and 2.303 is a constant; the molar extinction coefficients (cm^−1^/(moles/litre)) of HbO_2_ and Hb were extracted from Prahl^21^; 0.002326 is the haemoglobin molar concentration defined by the ratio of the haemoglobin concentration (150 gm/litre) to gram molecular weight (64,500). The combined HbO_2_ and Hb absorbance is then *0*.*85 aHbO*_*2*_ + *0*.*15 aHb* based on the average of the HbO_2_ proportions in arteries (95%) and veins (75%)^22^. For the open-field melanopsin photoreceptor isolating condition, the penumbral L- and M-cone contrast was ≤0.6% and the penumbral S-cone contrast was 0.5%. These contrast levels are below blue-yellow (S-cone) thresholds, but above the red-green (+L − M cone) chromatic thresholds^8^ and so we generated temporal white noise to desensitise these penumbral cones (Fig. 1b). This noise is a form of visual adaptation because the presentation is prolonged (2 s) and it is not simultaneous with the stimulus, as is often the case in many visual masking experiments.

Temporal white noise (TWN) randomly modulates the S-cone, M-cone, L-cone and Rod photoreceptor excitations (40% Michelson contrast)^23,24^ without changing the melanopsin photoreceptor excitation. The noise is generated in the frequency domain by assigning fixed amplitudes to all frequencies between 0 and 30 Hz and randomly varying phase (0–359°). The inverse Fast Fourier Transform (FFT) results in 1024 noise samples evenly distributed within a 1 s stimulus window (Fig. 1b). In the temporal domain, this results in randomly varied S-cone, M-cone, L-cone and Rod photoreceptor excitations that are Gaussian distributed around the mean adaptation level. Noise amplitudes in the frequency domain are therefore equal (constant power spectral density) for all temporal frequencies up to the 30 Hz cut-off frequency (Fig. 1c).

### Experimental design

#### Human psychophysical measurements

Detection thresholds were measured in response to 1000 ms duration incremental pulses on the white, yellowish-pink and orange fields. Temporal contrast sensitivity functions were measured on an orange adapting stimulus field for 1000 ms duration sinusoidal stimuli for frequencies ≥1 Hz and the reciprocal of the frequency when <1 Hz. Thresholds were estimated using a 2-yes 1-no psychophysical procedure with a double random alternating staircase with either penumbral cone silencing temporal white noise (Fig. 1d) or without noise (Fig. 1e), for three photoreceptor combinations: (1) L- and M-cones modulated in-phase to produce a cone luminance (L + M) signal with no change in excitation of the rods or melanopsin, (2) S-cone modulation with no change in the excitation of melanopsin, rods and the L- and M-cones, and (3) melanopsin with no change in the excitation of the rods and three cone types. A tone signalled the start of each trial and observers recorded their responses via a button press on a hand-held game pad. A 2000 ms inter-stimulus interval preceded onset of the 1000 ms sinusoidal stimulus waveform (80% stimulus probability on each trial). Visual detection and temporal contrast thresholds were measured in separate sessions. Temporal frequency, photoreceptor type and noise condition (with or without noise) were randomly ordered. Thresholds were defined as the average of the last 6 reversals of the staircase procedure (final step sizes = 0.009 log unit) and computed from a minimum of three repeats. The critical flicker frequency (CFF) was estimated for each photoreceptor condition using a method of adjustment at 17% Michelson contrast for adaptation levels spanning 200 Td to 5000 Td on the orange field. The 2000 ms inter-stimulus interval included noise (melanopsin conditions) or no noise (L + M and S-cone conditions).

The colour appearance of melanopsin signalling was quantified with a psychophysical temporal matching technique^18^ (Fig. 1f) whereby an incremental melanopsin stimulus (without changing the cone and rod photoreceptor excitations using silent substitution) of 7% Weber contrast on the white field, 22% on the yellowish-pink field and 24% on the orange field was metamerically matched in appearance to a cone-mediated visual stimulus that required observers to independently adjust the cone signals along three visual dimensions (without changing the melanopsin or rod photoreceptor excitation) to modulate L + M cone inputs for cone luminance, S/(L + M) along the putative blue-yellow dimension and L/(L + M)] along the putative red-green dimension. In the 3D colour spaces (Fig. 1f) these three directions are represented schematically as characteristic changes in the appearance of the lights depending if the changes increase (+) or decrease (−) the cone photoreceptor excitations: For luminance (L + M) the changes are brighter (+) or dimmer (−); for S/(L + M) the changes appear as purple (+) or lime (−); for L/(L + M)] the changes appear as magenta (+) or cyan (−) (Fig. 1f). The observers freely toggled between a melanopsin epoch and a cone epoch when setting their metameric match using the hand-held game pad.

In order to evaluate the effect of different relative cone excitations on melanopsin hue percepts, the chromaticities of the 2000 photopic Td adapting fields were white, yellowish-pink and orange. The combination of cone inputs that match the melanopsin percept is unknown, so during the practice trials the observers completed their matches under instruction to try alternate strategies to find the metameric match, including to modulate any combination of the three dimensions in any order, by changing the luminance component before altering the chromatic dimensions, and by changing a chromatic dimension and then altering luminance. During data collection they used their preferred approach. The melanopsin (or cone stimulus) was presented for 1000 ms every 1500 ms until the observer toggled to the alternate stimulus epoch; noise was continuously present during the inter-stimulus interval. At the metameric match the melanopsin and cone signals are indistinguishable and therefore the colour appearance of melanopsin directed stimuli are specified in terms of cone signalling without relying on a subjective colour label to describe how the melanopsin stimulus looks. Data for each observer were the minimum of 10 colour matches.

#### Pupil flicker responses

The pupil response was recorded under the same adaptation and viewing conditions as the psychophysical experiments using the photoreceptor modulation combinations with and without noise for a 1 Hz sinusoidal stimulus chosen because it is near the peak sensitivity of the human pupil flicker response^25^. Pupil analyses followed our established procedures^26,27^. For the pupil recordings, the 1 Hz stimuli were presented in 1 s epochs separated by a 1 ms blank interval and repeated 40 times during each 40.04 s recording sequence. To avoid onset artefacts, the responses to the first 10 stimulus cycles were discarded. Pupil responses were extracted after artefact rejection for blinks using Fourier transforms in each 1 s epoch to compute the mean pupil signal from the individual epochs using MATLAB (R2016b, Mathworks, USA). Data were measured during the day to minimise the influence of circadian variation on melanopsin-mediated pupil responses^26^; each participant was scheduled at the same test time for their test sessions on different days.

#### Entopic percept of the retinal blood vessels

A four point rating of the entopic appearance of the retinal blood vessels (0 = No spatial structure; 1 = any spatial structure; 2 = faint Purkinje tree; 3 = strong Purkinje tree)^10^, was completed for two temporal frequencies: the transition frequency 4 Hz at which participants might first notice the appearance of the Purkinje tree, and at 10 Hz, the frequency at which the entopic phenomenon is most apparent^10^. Ratings were obtained at the highest and lowest adaptation levels (5000 and 2000 Td), with and without temporal white noise, and for the 1000 ms incremental pulse.

### Data and materials availability

The datasets generated during and/or analysed during the current study are available from the corresponding author on reasonable request.

## Results

**Figure 2:**
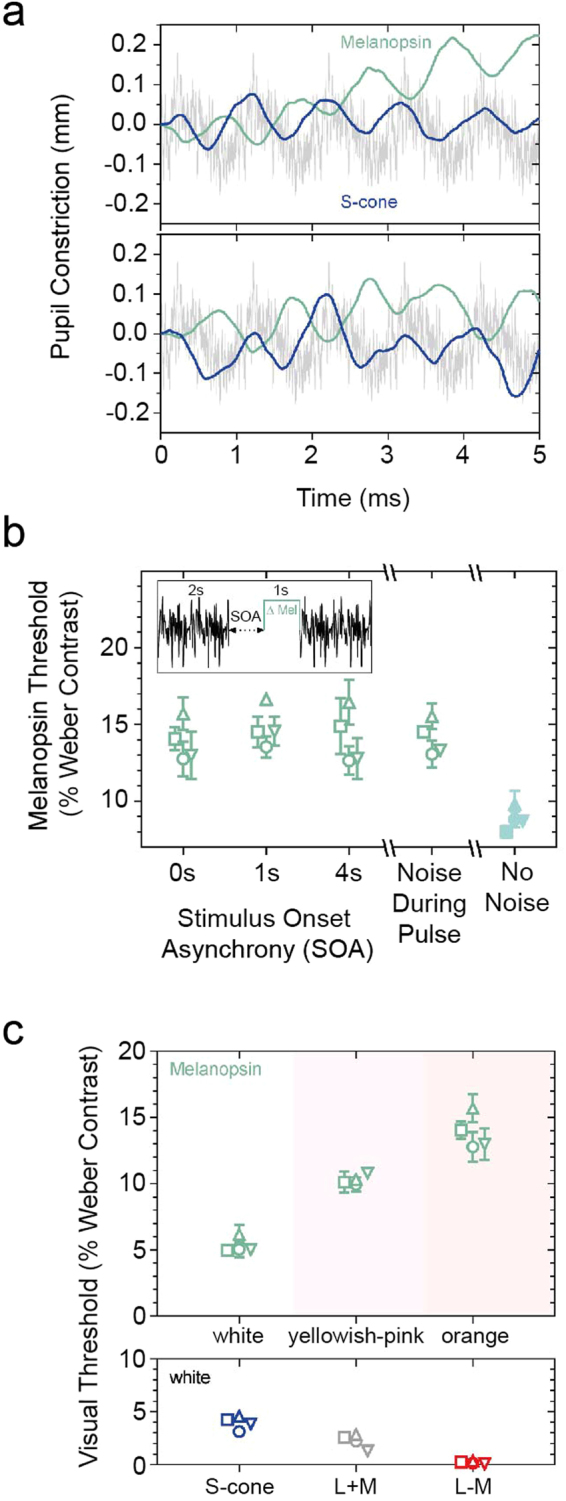
Photoreception for melanopsin directed stimuli measured under penumbral cone silencing conditions. (**a**) Melanopsin pupil flicker responses in penumbral cone silencing temporal white noise are opponent to the paradoxical S-cone pupil response that dilates with increasing irradiance (n = 2 observers; 227°, 202° phase difference). (**b**) Visual increment thresholds (n = 4 observers; mean ± SEM) for melanopsin (orange field, 2000 photopic Td) measured as a function of the time (stimulus onset asynchrony, SOA) between offset of the 40% noise and the onset of the melanopsin directed pulse, with 15% penumbral cone noise synchronous with the melanopsin pulse (noise during pulse), and a condition without noise (no noise). (**c**) Visual increment thresholds (n = 4 observers; mean ± SEM) for melanopsin with adapting stimulus fields of different relative cone excitations that appeared white, yellowish-pink or orange, and for the three cone directed visual stimulations measured on a white field.

**Figure 3:**
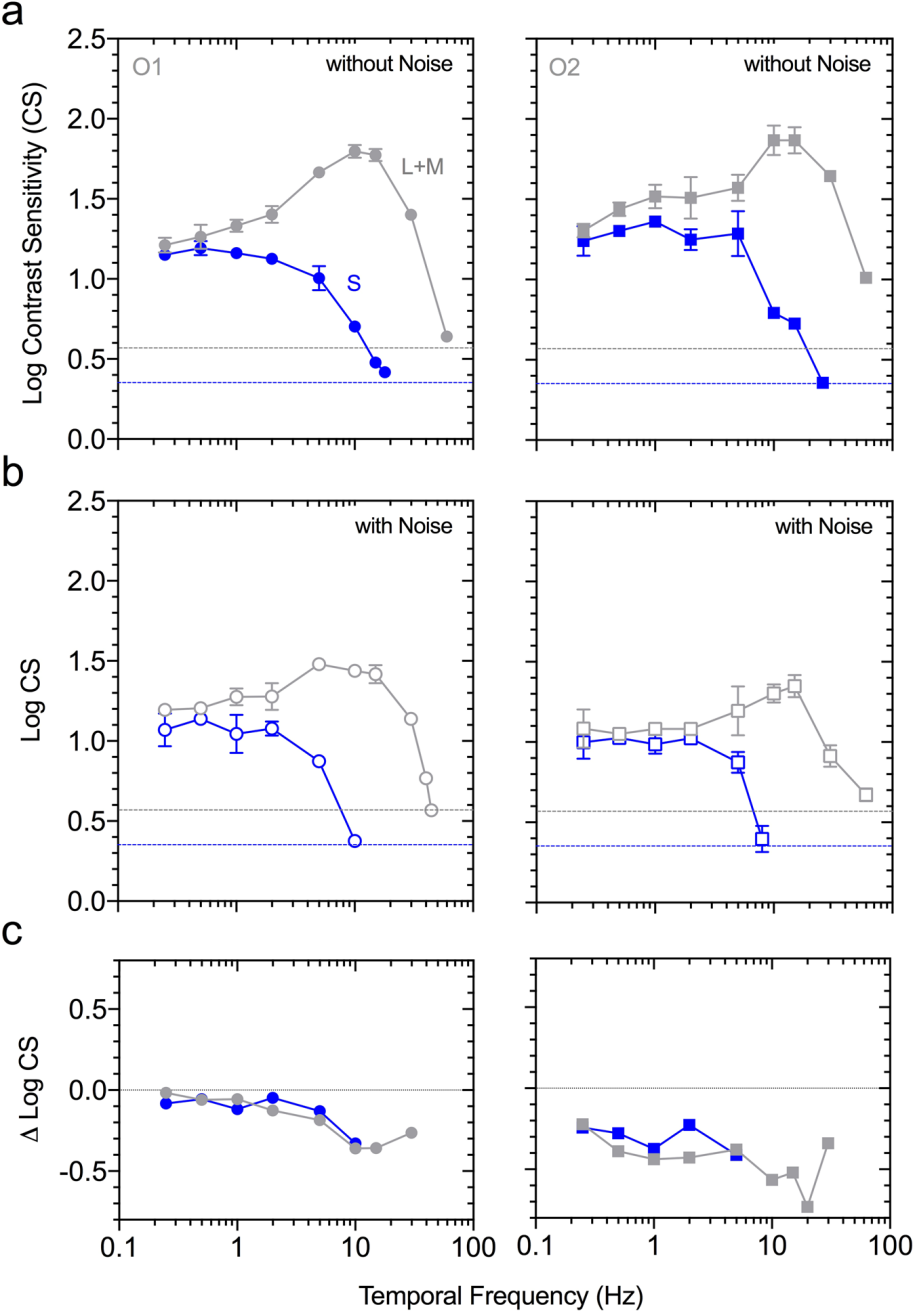
Desensitising cone signals with temporal white noise. (**a**) Luminance (L + M cone) and S-cone temporal contrast sensitivity functions (mean ± SEM) for two participants (left panels, Observer O1; right panels, Observer O2) measured at 2000 photopic Td (no change in excitation of the other photoreceptor types) in the control condition without noise or (**b**) with penumbral cone silencing temporal white noise that modulates the S-cone, M-cone, L-cone and Rod photoreceptor excitations without changing the melanopsin photoreceptor excitation. Temporal contrast sensitivity is bandpass for luminance and low-pass for S-cones. Horizontal lines show the gamut limits for the L + M cone (grey line) and S-cone stimuli (blue line). (**c**) The visuogram shows the white noise attenuation of cone sensitivity.

A known neurophysiological correlate of the ipRGC response property in humans is the characteristic opponent melanopsin and S-cone pupil response^9,28^ and we confirm that melanopsin inputs to the pupil response are unaffected by temporal white noise (Fig. 2a). The white noise frequency spectrum attenuates cone (L + M) luminance and S-cone mediated temporal contrast sensitivity (Fig. 3) and desensitises penumbral cones to allow for the direct measurement of melanopsin inputs to image-forming vision. The white noise adapts the rod and cone signals for at least 4 s from noise offset, with minimal influence on the melanopsin directed threshold when presented synchronously (Fig. 2b). Visual thresholds for melanopsin depend on the saturation of the adapting light and are higher than thresholds mediated by each of the three cone dimensions (Fig. 2c).

**Figure 4:**
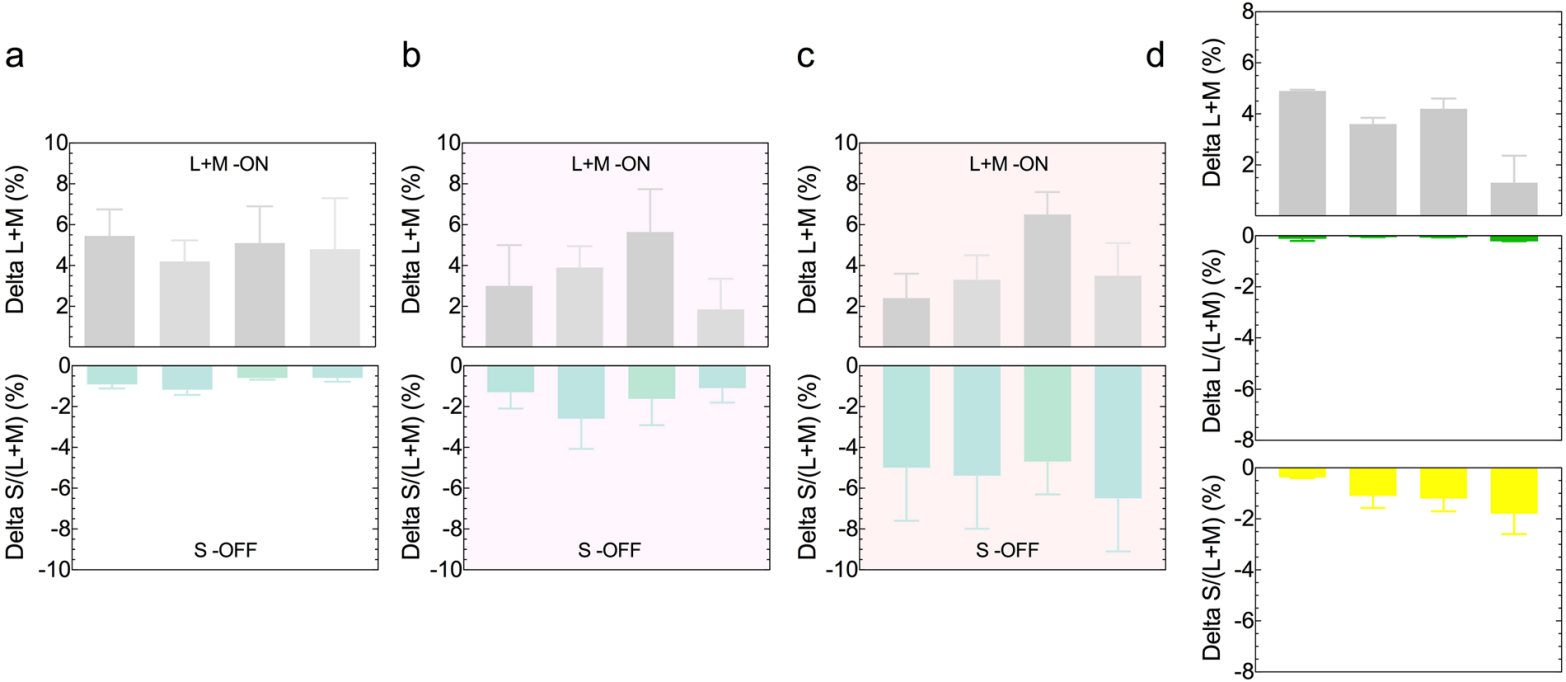
Melanopsin photoreception is analogous to an increment in cone luminance [L + M] and a decrement in S-cone excitation [S/(L + M)] with white (**a**), yellowish-pink (**b**) and orange adapting stimulus fields (**c**). The melanopsin colour signal (7% Weber contrast; white field) shows a −L + M [Delta L/L + M] penumbral cone intrusion in the absence of temporal white noise (**d**). Data in each panel are for four participants (mean ± SEM) measured at 2000 photopic Td.

We successfully quantified melanopsin contributions to colour vision in terms of equivalent cone signals. A unique and salient melanopsin colour percept is observed when penumbral cones are desensitised. This melanopsin percept corresponds to an increase in cone luminance [L + M] and a decrease in S-cone photoreceptor excitation (Fig. 4a), indicating melanopsin photoreception has the equivalent consequence of the colour opponent S-OFF L + M-ON response property of ipRGCs in human vision. Penumbral L- and M-cone intrusion is signified by the presence of an −L + M cone contribution (Delta L/L + M) to the metameric colour match (Fig. 4d). When the relative cone photoreceptor excitations change with the spectral properties of the illuminant, similar to that experienced during periodic variations in the light-dark environment, the melanopsin response property is stable (Fig. 4b,c).

**Figure 5:**
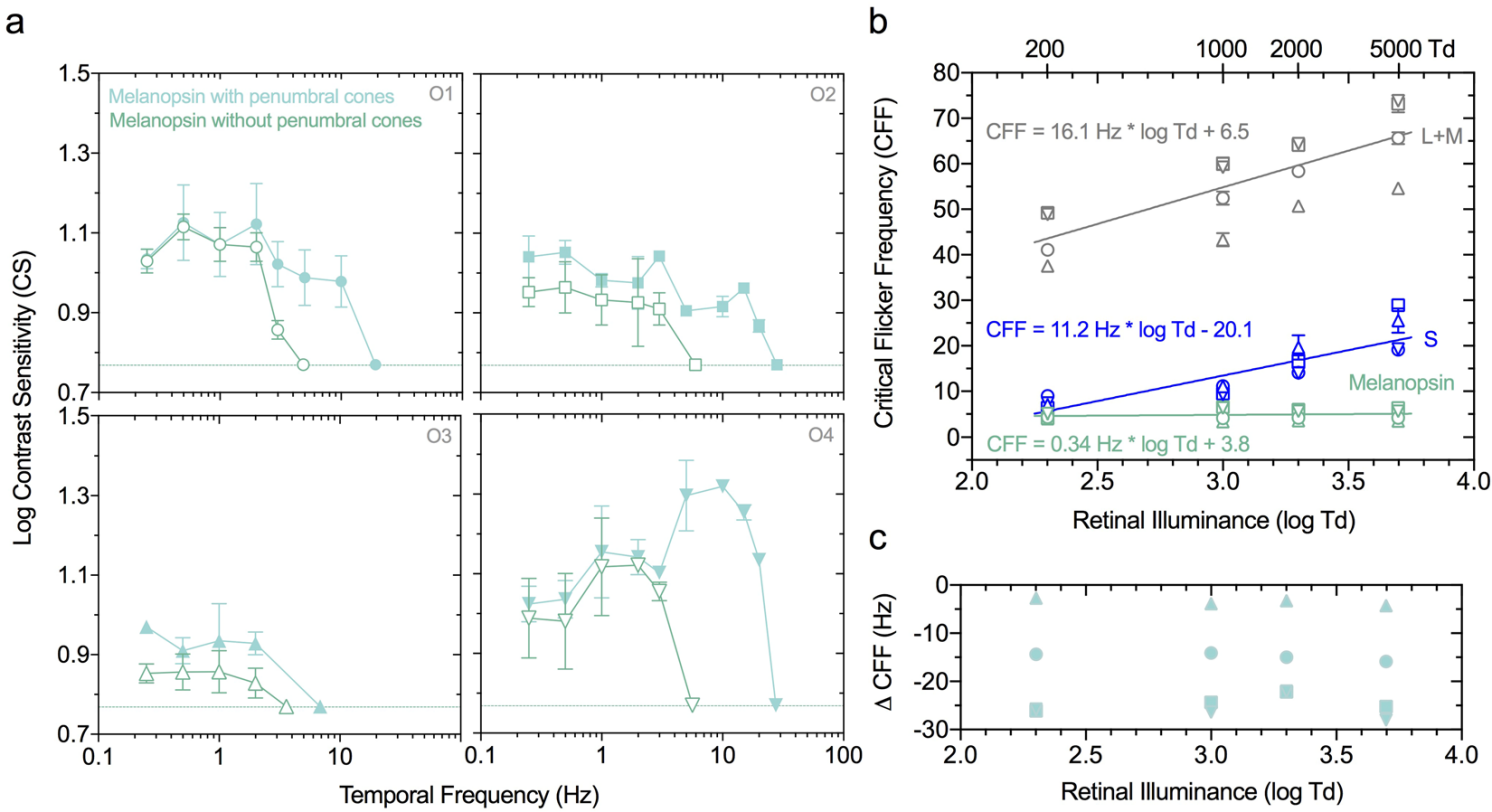
Photopic melanopsin-mediated visual temporal contrast sensitivity (**a**) with penumbral cone silencing temporal white noise (unfilled symbols) and without noise (filled symbols) for four observers (O1-O4: Mean ± SEM). The horizontal lines indicate the instrument gamut limit for melanopsin isolation. (**b**) Ferry-Porter Law behavior for melanopsin (with white noise), L + M cone luminance (without noise) and S-cones (without noise) (n = 4 observers; mean ± SEM). Lines show the best-fitting linear Ferry-Porter functions to the average data. (**c**) Penumbral cone critical flicker frequency for the melanopsin directed stimuli measured without white noise (n = 4 observers).

Since the intrinsic melanopsin photoresponse displays a slow and sustained temporal response in single-cell recordings^3^, we sought to determine its perceptual correlate using our penumbral cone silencing method. All participants reported a zero entopic percept (no spatial structure) with the 4 Hz and 10 Hz melanopsin stimuli (with and without white noise) at the highest and lowest adaptation levels (5000 and 2000 Td). With the 1 s melanopsin pulse, all participants reported a zero entopic percept with and without white noise at 2000 Td; two observers reported the appearance of any spatial structure (rating = 1) at 5000 Td (without white noise). For melanopsin directed stimuli, visual contrast sensitivity is low pass with a cut-off frequency of 4.9 ± 1.1 Hz (Fig. 5a, unfilled symbols). Linear increases in high temporal frequency cut-offs for cone luminance (16.1 Hz increase per log unit, p = 0.004) and S-cone signalling (11.2 Hz increase per log unit, p = 0.06) are as expected, in accordance with the Ferry-Porter law, however melanopsin signalling is relatively invariant with increasing light level (0.34 Hz per log unit, p = 0.32) (Fig. 5b). The detection of temporal frequencies beyond the resolution limit of melanopsin highlights the significance of white noise for desensitising penumbral cone signalling (Fig. 5a, filled symbols) in the photopic range (Fig. 5c).

## Discussion

Conscious visual percepts accompanied by melanopsin directed stimulation parallel the observed S-OFF/L + M-ON response property identified in anatomical studies of the macaque LGN^3^ and so melanopsin might form an independent visual dimension (Figs 2b,c; 4). Even though the visual threshold for melanopsin directed stimulation was higher than for cone directed modulations, melanopsin contributions to vision mean that its signalling can interact with outer retinal photoreceptor signals to shape rod and cone vision, as evident in mice^29^. Defining these yet to be explored interactions with rod and cone mediated vision will be critical for generating a complete picture of human vision. There is a single case report of a blind woman (87 years of age; autosomal-dominant cone–rod dystrophy) who could detect the presence of an intense 480 nm light near the peak sensitivity of melanopsin, but not at other wavelengths^30^. Any changes in melanopsin function in people with retinal, neurological or chronobiology disorders might also affect rod and cone sensitivity^31^.

Melanopsin inputs to vision^6,7,32,33^ and the pupil light reflex^4,9,28,34^ could have different thresholds and photoreceptor contributions in the intact visual system, as studied here, compared to single and multi-cell recordings and in transgenic rodless/coneless models, and require a different number of cells to drive functional responses^35^. The M1 ipRGCs have a low photopigment density, onset latencies as long as 200 ms, yet seem capable of signalling single photon absorptions through increases in spike rate. Furthermore, ipRGCs robustly sustain synaptically mediated extrinsic light responses under steady illumination when conventional ganglion cells seldom respond^3,36^. These extrinsic inputs increase the temporal frequency response and sensitivity of ipRGCs beyond its intrinsic capability^36^ and may influence visual coding under steady illumination. We observe under steady illumination that cone-mediated vision is more sensitive (Fig. 2c) with higher temporal contrast responses than the intrinsic visual response to melanopsin directed stimulation (Figs 3 and 5). The normalisation of chromatic vision to an average white value in the natural environment^37^ leads to contrast thresholds being contingent on the adapting chromaticity^38^. Thresholds for melanopsin directed stimulation appear to show a similar relationship (Fig. 2c), indicating the relative adaptation state of the cones may be important for melanopsin contributions to vision. In mice, melanopsin signalling can also adjust cone pathway sensitivity under naturalistic viewing conditions^29^.

Our inference for melanopsin contributions to conscious vision are dependent on the successful separation of any image forming responses arising from the theoretical activation of melanopsin (if they are to exist) from potential response artefacts arising from failure to completely silence the rods and cones. As such, the factors influencing the theoretical isolation of melanopsin-mediated photoreception must be considered. Relevant to the method of silent substitution, the largest source of variation in the estimates of the photoreceptor spectral sensitivities of normal trichromats^39^ is optical lens attenuation and so we conducted observer calibrations with the intention to minimise individual differences between participants and the theoretical standard observer L + M cone luminous efficiency function. By doing so, each observer had a personalised estimate of their cone luminous efficiency that was used to normalise the relative photoreceptor excitations of the S-cones, rods and melanopsin. The issue of how to address penumbral cone intrusion has not been previously resolved. Although umbral cones deep within the vessel shadows are not represented in the cortex, the penumbral cone signals are^40^. In this experiment, none of the observers reported any entopic phenomenon with the penumbral cone silencing temporal white noise. Previous research showed that an entopic Purkinje tree is visible at higher temporal frequencies (≥ 4 Hz) under steady state viewing conditions without macular blocking^10^, at light levels ~7x higher than our mean adaptation level. A recent study^5^ implied that melanopsin could ‘see’ high contrast stimuli atypical of those encountered in the natural environment, but did not establish whether the visual percept was a result of the processing of ipRGC signals in area V1, or along the visual pathway due to cone interactions; the eccentricity dependent increase in retinal vessel diameter resulted in their melanopsin directed stimulus (400% contrast; 64° outer diameter)^5^ producing about a 5% L- and M-cone contrast, more than 50x the chromatic red-green (+L − M) threshold obtained with our set-up. Here we estimated that our theoretical melanopsin directed stimuli produced penumbral cone contrast signals that were sufficient to generate threshold responses via a cone-mediated process. To limit this intrusion, we developed a temporal white noise adaptation method (Fig. 1b) to desensitise the L-, M- and S-cones and rods (Fig. 3c) and which had minimal influence on thresholds for melanopsin directed stimuli to allow the application of lower contrast stimuli (Fig. 2b), and in which the opponent melanopsin and S-cone pupil response was still measurable (Fig. 2a).

Scattered light into the peripheral visual field could lead to the unwanted intrusion of more sensitive (rod and cone) visual processes during melanopsin directed stimulation. It would be present during all measurements and so any contribution of scatter to cone luminance signalling would be accounted for during the observer calibration. To limit any potential influence of light scatter at the outer edge of the 30° diameter stimulus field, observers were instructed to make all psychophysical judgments based on perceptual changes occurring near the inner edge of the stimulus (i.e. 5.25° from the fixation marker). Rod isolated thresholds were not measurable at the photopic adaptation levels. Eye movements have little effect on the temporal contrast sensitivity function^41^ whereas field size does. Low temporal frequency (<8 Hz) contrast sensitivity is reduced by more than 10% with large field diameters (65° vs. 2°^41,42^); and so the detection mechanism(s) may be less sensitive to artefacts arising from light scatter at the outer edge of the large 30° diameter stimulus field than they would be had smaller fields been used. If scattered light contributed to the critical flicker fusion frequency estimates for the melanopsin directed stimuli (Fig. 5b), the slope of the Ferry-Porter function would be closer to that of the two cone functions, but it was not.

Spectral distributions in the natural environment show the largest variations in cone luminance and then blue-yellow chromatic information^43^, factors that were thought to be critical for the evolution of trichromatic colour vision^44^. Melanopsin, an ancient photopigment with different lineage and function compared to, and predating, photosensory cone opsins^45^, provided the primitive visual systems of early vertebrates with the capacity to signal illumination changes. With evolution of cone and rod opsins capable of signalling transient changes in image contrast, we hypothesize that melanopsin-mediated human vision may complement classical cone vision to incur an ecological advantage for colour vision, in addition to that for irradiance coding^6,7^. Our findings of melanopsin hue perception (Fig. 4a) and slow temporal integration that is invariant with light level (Fig. 5b) are in line with the notion that ipRGCs might support a long-term estimate of the chromaticity of the ambient illumination to maintain the stable perceived colour of objects (i.e. colour constancy) despite continually changing environmental spectral light distributions (Fig. 4b,c). Trichromatic theory satisfactorily describes photopic colour vision in the central retina^46^ where there are no melanopsin cells^3,47,48^, but not in the peripheral retina that requires a tetrachromatic model^8^; our observations may signify a role for melanopsin-containing ipRGCs as the fifth photoreceptor type for human vision.

## Electronic supplementary material

Supplementary information
