# Supplementary material for: Melanopsin photoreception contributes to human visual detection, temporal and colour processing
Source: Sci Rep. 2018 Mar 1;8:3842. doi: 10.1038/s41598-018-22197-w (PMC5832793; doi:10.1038/s41598-018-22197-w)
Supplement: Supplementary file 1 — Supplementary information [file 41598_2018_22197_MOESM1_ESM.docx]

**Melanopsin photoreception contributes to human visual detection,**

**temporal and colour processing**

Andrew J. Zele,^1,2 *^ Beatrix Feigl,^1,3,4^ Prakash Adhikari,^1,2^

Michelle L. Maynard,^1,2,3^ Dingcai Cao^5^

^1^ Institute of Health and Biomedical Innovation, ^2^ School of Optometry and Vision Science,

^3^ School of Biomedical Sciences, Queensland University of Technology (QUT), Brisbane, Australia.

^4^ Queensland Eye Institute, Brisbane, Australia.

^5^ Department of Ophthalmology and Visual Sciences, University of Illinois at Chicago, USA.

**Supplementary Table S1.** Relative spectral outputs of the 5 primary lights for the CIE 1964 10° Standard Observer as shown in Figure 1a.

| Primary Light (Irradiance; Watts.cm^-2.^s^-1^) | | | | | | | | | | |
| --- | --- | --- | --- | --- | --- | --- | --- | --- | --- | --- |
|  | Orange appearing adapting background field (2000 Td) | | | | | 17% Weber contrast increment in melanopsin excitation | | | | |
| **nm** | Blue | Cyan | Green | Amber | Red | Blue | Cyan | Green | Amber | Red |
| 400 | 0.000000 | 0.000000 | 0.000000 | 0.000000 | 0.000000 | 0.000000 | 0.000000 | 0.000000 | 0.000000 | 0.000000 |
| 401 | 0.000000 | 0.000000 | 0.000000 | 0.000000 | 0.000000 | 0.000000 | 0.000000 | 0.000000 | 0.000000 | 0.000000 |
| 402 | 0.000000 | 0.000000 | 0.000000 | 0.000000 | 0.000000 | 0.000000 | 0.000000 | 0.000000 | 0.000000 | 0.000000 |
| 403 | 0.000000 | 0.000000 | 0.000000 | 0.000000 | 0.000000 | 0.000000 | 0.000000 | 0.000000 | 0.000000 | 0.000000 |
| 404 | 0.000000 | 0.000000 | 0.000000 | 0.000000 | 0.000000 | 0.000000 | 0.000000 | 0.000000 | 0.000000 | 0.000000 |
| 405 | 0.000000 | 0.000000 | 0.000000 | 0.000000 | 0.000000 | 0.000000 | 0.000000 | 0.000000 | 0.000000 | 0.000000 |
| 406 | 0.000000 | 0.000000 | 0.000000 | 0.000000 | 0.000000 | 0.000000 | 0.000000 | 0.000000 | 0.000000 | 0.000000 |
| 407 | 0.000000 | 0.000000 | 0.000000 | 0.000000 | 0.000000 | 0.000000 | 0.000000 | 0.000000 | 0.000000 | 0.000000 |
| 408 | 0.000000 | 0.000000 | 0.000000 | 0.000000 | 0.000000 | 0.000000 | 0.000000 | 0.000000 | 0.000000 | 0.000000 |
| 409 | 0.000000 | 0.000000 | 0.000000 | 0.000000 | 0.000000 | 0.000000 | 0.000000 | 0.000000 | 0.000000 | 0.000000 |
| 410 | 0.000000 | 0.000000 | 0.000000 | 0.000000 | 0.000000 | 0.000000 | 0.000000 | 0.000000 | 0.000000 | 0.000000 |
| 411 | 0.000000 | 0.000000 | 0.000000 | 0.000000 | 0.000000 | 0.000000 | 0.000000 | 0.000000 | 0.000000 | 0.000000 |
| 412 | 0.000000 | 0.000000 | 0.000000 | 0.000000 | 0.000000 | 0.000000 | 0.000000 | 0.000000 | 0.000000 | 0.000000 |
| 413 | 0.000000 | 0.000000 | 0.000000 | 0.000000 | 0.000000 | 0.000000 | 0.000000 | 0.000000 | 0.000000 | 0.000000 |
| 414 | 0.000000 | 0.000000 | 0.000000 | 0.000000 | 0.000000 | 0.000000 | 0.000000 | 0.000000 | 0.000000 | 0.000000 |
| 415 | 0.000000 | 0.000000 | 0.000000 | 0.000000 | 0.000000 | 0.000000 | 0.000000 | 0.000000 | 0.000000 | 0.000000 |
| 416 | 0.000000 | 0.000000 | 0.000000 | 0.000000 | 0.000000 | 0.000000 | 0.000000 | 0.000000 | 0.000000 | 0.000000 |
| 417 | 0.000000 | 0.000000 | 0.000000 | 0.000000 | 0.000000 | 0.000000 | 0.000000 | 0.000000 | 0.000000 | 0.000000 |
| 418 | 0.000000 | 0.000000 | 0.000000 | 0.000000 | 0.000000 | 0.000000 | 0.000000 | 0.000000 | 0.000000 | 0.000000 |
| 419 | 0.000000 | 0.000000 | 0.000000 | 0.000000 | 0.000000 | 0.000000 | 0.000000 | 0.000000 | 0.000000 | 0.000000 |
| 420 | 0.000000 | 0.000000 | 0.000000 | 0.000000 | 0.000000 | 0.000000 | 0.000000 | 0.000000 | 0.000000 | 0.000000 |
| 421 | 0.000000 | 0.000000 | 0.000000 | 0.000000 | 0.000000 | 0.000000 | 0.000000 | 0.000000 | 0.000000 | 0.000000 |
| 422 | 0.000000 | 0.000000 | 0.000000 | 0.000000 | 0.000000 | 0.000000 | 0.000000 | 0.000000 | 0.000000 | 0.000000 |
| 423 | 0.000000 | 0.000000 | 0.000000 | 0.000000 | 0.000000 | 0.000000 | 0.000000 | 0.000000 | 0.000000 | 0.000000 |
| 424 | 0.000000 | 0.000000 | 0.000000 | 0.000000 | 0.000000 | 0.000000 | 0.000000 | 0.000000 | 0.000000 | 0.000000 |
| 425 | 0.000000 | 0.000000 | 0.000000 | 0.000000 | 0.000000 | 0.000000 | 0.000000 | 0.000000 | 0.000000 | 0.000000 |
| 426 | 0.000000 | 0.000000 | 0.000000 | 0.000000 | 0.000000 | 0.000000 | 0.000000 | 0.000000 | 0.000000 | 0.000000 |
| 427 | 0.000000 | 0.000000 | 0.000000 | 0.000000 | 0.000000 | 0.000000 | 0.000000 | 0.000000 | 0.000000 | 0.000000 |
| 428 | 0.000000 | 0.000000 | 0.000000 | 0.000000 | 0.000000 | 0.000000 | 0.000000 | 0.000000 | 0.000000 | 0.000000 |
| 429 | 0.000000 | 0.000000 | 0.000000 | 0.000000 | 0.000000 | 0.000000 | 0.000000 | 0.000000 | 0.000000 | 0.000000 |
| 430 | 0.000000 | 0.000000 | 0.000000 | 0.000000 | 0.000000 | 0.000000 | 0.000000 | 0.000000 | 0.000000 | 0.000000 |
| 431 | 0.000000 | 0.000000 | 0.000000 | 0.000000 | 0.000000 | 0.000000 | 0.000000 | 0.000000 | 0.000000 | 0.000000 |
| 432 | 0.000000 | 0.000000 | 0.000000 | 0.000000 | 0.000000 | 0.000000 | 0.000000 | 0.000000 | 0.000000 | 0.000000 |
| 433 | 0.000000 | 0.000000 | 0.000000 | 0.000000 | 0.000000 | 0.000000 | 0.000000 | 0.000000 | 0.000000 | 0.000000 |
| 434 | 0.000000 | 0.000000 | 0.000000 | 0.000000 | 0.000000 | 0.000000 | 0.000000 | 0.000000 | 0.000000 | 0.000000 |
| 435 | 0.000000 | 0.000000 | 0.000000 | 0.000000 | 0.000000 | 0.000000 | 0.000000 | 0.000000 | 0.000000 | 0.000000 |
| 436 | 0.000000 | 0.000000 | 0.000000 | 0.000000 | 0.000000 | 0.000000 | 0.000000 | 0.000000 | 0.000000 | 0.000000 |
| 437 | 0.000000 | 0.000000 | 0.000000 | 0.000000 | 0.000000 | 0.000000 | 0.000000 | 0.000000 | 0.000000 | 0.000000 |
| 438 | 0.000000 | 0.000000 | 0.000000 | 0.000000 | 0.000000 | 0.000000 | 0.000000 | 0.000000 | 0.000000 | 0.000000 |
| 439 | 0.000000 | 0.000000 | 0.000000 | 0.000000 | 0.000000 | 0.000000 | 0.000000 | 0.000000 | 0.000000 | 0.000000 |
| 440 | 0.000000 | 0.000000 | 0.000000 | 0.000000 | 0.000000 | 0.000000 | 0.000000 | 0.000000 | 0.000000 | 0.000000 |
| 441 | 0.000000 | 0.000000 | 0.000000 | 0.000000 | 0.000000 | 0.000000 | 0.000000 | 0.000000 | 0.000000 | 0.000000 |
| 442 | 0.000000 | 0.000000 | 0.000000 | 0.000000 | 0.000000 | 0.000000 | 0.000000 | 0.000000 | 0.000000 | 0.000000 |
| 443 | 0.000000 | 0.000000 | 0.000000 | 0.000000 | 0.000000 | 0.000000 | 0.000000 | 0.000000 | 0.000000 | 0.000000 |
| 444 | 0.000000 | 0.000000 | 0.000000 | 0.000000 | 0.000000 | 0.000000 | 0.000000 | 0.000000 | 0.000000 | 0.000000 |
| 445 | 0.000001 | 0.000000 | 0.000000 | 0.000000 | 0.000000 | 0.000000 | 0.000000 | 0.000000 | 0.000000 | 0.000000 |
| 446 | 0.000040 | 0.000000 | 0.000000 | 0.000000 | 0.000000 | 0.000013 | 0.000000 | 0.000000 | 0.000000 | 0.000000 |
| 447 | 0.000090 | 0.000000 | 0.000000 | 0.000000 | 0.000000 | 0.000028 | 0.000000 | 0.000000 | 0.000000 | 0.000000 |
| 448 | 0.000137 | 0.000000 | 0.000000 | 0.000000 | 0.000000 | 0.000043 | 0.000000 | 0.000000 | 0.000000 | 0.000000 |
| 449 | 0.000201 | 0.000000 | 0.000000 | 0.000000 | 0.000000 | 0.000063 | 0.000000 | 0.000000 | 0.000000 | 0.000000 |
| 450 | 0.000374 | 0.000000 | 0.000000 | 0.000000 | 0.000000 | 0.000118 | 0.000000 | 0.000000 | 0.000000 | 0.000000 |
| 451 | 0.000680 | 0.000000 | 0.000000 | 0.000000 | 0.000000 | 0.000214 | 0.000000 | 0.000000 | 0.000000 | 0.000000 |
| 452 | 0.001021 | 0.000000 | 0.000000 | 0.000000 | 0.000000 | 0.000321 | 0.000000 | 0.000000 | 0.000000 | 0.000000 |
| 453 | 0.001314 | 0.000000 | 0.000000 | 0.000000 | 0.000000 | 0.000414 | 0.000000 | 0.000000 | 0.000000 | 0.000000 |
| 454 | 0.001543 | 0.000000 | 0.000000 | 0.000000 | 0.000000 | 0.000486 | 0.000000 | 0.000000 | 0.000000 | 0.000000 |
| 455 | 0.001766 | 0.000000 | 0.000000 | 0.000000 | 0.000000 | 0.000556 | 0.000000 | 0.000000 | 0.000000 | 0.000000 |
| 456 | 0.001897 | 0.000000 | 0.000000 | 0.000000 | 0.000000 | 0.000597 | 0.000000 | 0.000000 | 0.000000 | 0.000000 |
| 457 | 0.001905 | 0.000000 | 0.000000 | 0.000000 | 0.000000 | 0.000600 | 0.000000 | 0.000000 | 0.000000 | 0.000000 |
| 458 | 0.001837 | 0.000000 | 0.000000 | 0.000000 | 0.000000 | 0.000578 | 0.000000 | 0.000000 | 0.000000 | 0.000000 |
| 459 | 0.001709 | 0.000000 | 0.000000 | 0.000000 | 0.000000 | 0.000538 | 0.000000 | 0.000000 | 0.000000 | 0.000000 |
| 460 | 0.001497 | 0.000000 | 0.000000 | 0.000000 | 0.000000 | 0.000471 | 0.000000 | 0.000000 | 0.000000 | 0.000000 |
| 461 | 0.001111 | 0.000000 | 0.000000 | 0.000000 | 0.000000 | 0.000350 | 0.000000 | 0.000000 | 0.000000 | 0.000000 |
| 462 | 0.000672 | 0.000000 | 0.000000 | 0.000000 | 0.000000 | 0.000212 | 0.000000 | 0.000000 | 0.000000 | 0.000000 |
| 463 | 0.000367 | 0.000000 | 0.000000 | 0.000000 | 0.000000 | 0.000116 | 0.000000 | 0.000000 | 0.000000 | 0.000000 |
| 464 | 0.000193 | 0.000000 | 0.000000 | 0.000000 | 0.000000 | 0.000061 | 0.000000 | 0.000000 | 0.000000 | 0.000000 |
| 465 | 0.000112 | 0.000000 | 0.000000 | 0.000000 | 0.000000 | 0.000035 | 0.000000 | 0.000000 | 0.000000 | 0.000000 |
| 466 | 0.000038 | 0.000003 | 0.000000 | 0.000000 | 0.000000 | 0.000012 | 0.000006 | 0.000000 | 0.000000 | 0.000000 |
| 467 | 0.000000 | 0.000019 | 0.000000 | 0.000000 | 0.000000 | 0.000000 | 0.000039 | 0.000000 | 0.000000 | 0.000000 |
| 468 | 0.000000 | 0.000019 | 0.000000 | 0.000000 | 0.000000 | 0.000000 | 0.000039 | 0.000000 | 0.000000 | 0.000000 |
| 469 | 0.000000 | 0.000017 | 0.000000 | 0.000000 | 0.000000 | 0.000000 | 0.000033 | 0.000000 | 0.000000 | 0.000000 |
| 470 | 0.000000 | 0.000002 | 0.000000 | 0.000000 | 0.000000 | 0.000000 | 0.000003 | 0.000000 | 0.000000 | 0.000000 |
| 471 | 0.000000 | 0.000022 | 0.000000 | 0.000000 | 0.000000 | 0.000000 | 0.000046 | 0.000000 | 0.000000 | 0.000000 |
| 472 | 0.000000 | 0.000078 | 0.000000 | 0.000000 | 0.000000 | 0.000000 | 0.000158 | 0.000000 | 0.000000 | 0.000000 |
| 473 | 0.000000 | 0.000142 | 0.000000 | 0.000000 | 0.000000 | 0.000000 | 0.000288 | 0.000000 | 0.000000 | 0.000000 |
| 474 | 0.000000 | 0.000199 | 0.000000 | 0.000000 | 0.000000 | 0.000000 | 0.000403 | 0.000000 | 0.000000 | 0.000000 |
| 475 | 0.000000 | 0.000240 | 0.000000 | 0.000000 | 0.000000 | 0.000000 | 0.000487 | 0.000000 | 0.000000 | 0.000000 |
| 476 | 0.000000 | 0.000276 | 0.000000 | 0.000000 | 0.000000 | 0.000000 | 0.000560 | 0.000000 | 0.000000 | 0.000000 |
| 477 | 0.000000 | 0.000299 | 0.000000 | 0.000000 | 0.000000 | 0.000000 | 0.000606 | 0.000000 | 0.000000 | 0.000000 |
| 478 | 0.000000 | 0.000354 | 0.000000 | 0.000000 | 0.000000 | 0.000000 | 0.000718 | 0.000000 | 0.000000 | 0.000000 |
| 479 | 0.000000 | 0.000478 | 0.000000 | 0.000000 | 0.000000 | 0.000000 | 0.000969 | 0.000000 | 0.000000 | 0.000000 |
| 480 | 0.000000 | 0.000690 | 0.000000 | 0.000000 | 0.000000 | 0.000000 | 0.001400 | 0.000000 | 0.000000 | 0.000000 |
| 481 | 0.000000 | 0.001010 | 0.000000 | 0.000000 | 0.000000 | 0.000000 | 0.002047 | 0.000000 | 0.000000 | 0.000000 |
| 482 | 0.000000 | 0.001504 | 0.000000 | 0.000000 | 0.000000 | 0.000000 | 0.003051 | 0.000000 | 0.000000 | 0.000000 |
| 483 | 0.000000 | 0.002181 | 0.000000 | 0.000000 | 0.000000 | 0.000000 | 0.004424 | 0.000000 | 0.000000 | 0.000000 |
| 484 | 0.000000 | 0.002944 | 0.000000 | 0.000000 | 0.000000 | 0.000000 | 0.005970 | 0.000000 | 0.000000 | 0.000000 |
| 485 | 0.000000 | 0.003620 | 0.000000 | 0.000000 | 0.000000 | 0.000000 | 0.007341 | 0.000000 | 0.000000 | 0.000000 |
| 486 | 0.000000 | 0.004140 | 0.000000 | 0.000000 | 0.000000 | 0.000000 | 0.008396 | 0.000000 | 0.000000 | 0.000000 |
| 487 | 0.000000 | 0.004504 | 0.000000 | 0.000000 | 0.000000 | 0.000000 | 0.009135 | 0.000000 | 0.000000 | 0.000000 |
| 488 | 0.000000 | 0.004740 | 0.000000 | 0.000000 | 0.000000 | 0.000000 | 0.009612 | 0.000000 | 0.000000 | 0.000000 |
| 489 | 0.000000 | 0.004844 | 0.000000 | 0.000000 | 0.000000 | 0.000000 | 0.009823 | 0.000000 | 0.000000 | 0.000000 |
| 490 | 0.000000 | 0.004760 | 0.000000 | 0.000000 | 0.000000 | 0.000000 | 0.009654 | 0.000000 | 0.000000 | 0.000000 |
| 491 | 0.000000 | 0.004428 | 0.000000 | 0.000000 | 0.000000 | 0.000000 | 0.008980 | 0.000000 | 0.000000 | 0.000000 |
| 492 | 0.000000 | 0.003845 | 0.000000 | 0.000000 | 0.000000 | 0.000000 | 0.007798 | 0.000000 | 0.000000 | 0.000000 |
| 493 | 0.000000 | 0.003079 | 0.000000 | 0.000000 | 0.000000 | 0.000000 | 0.006245 | 0.000000 | 0.000000 | 0.000000 |
| 494 | 0.000000 | 0.002263 | 0.000000 | 0.000000 | 0.000000 | 0.000000 | 0.004590 | 0.000000 | 0.000000 | 0.000000 |
| 495 | 0.000000 | 0.001514 | 0.000000 | 0.000000 | 0.000000 | 0.000000 | 0.003070 | 0.000000 | 0.000000 | 0.000000 |
| 496 | 0.000000 | 0.000927 | 0.000000 | 0.000000 | 0.000000 | 0.000000 | 0.001879 | 0.000000 | 0.000000 | 0.000000 |
| 497 | 0.000000 | 0.000531 | 0.000000 | 0.000000 | 0.000000 | 0.000000 | 0.001078 | 0.000000 | 0.000000 | 0.000000 |
| 498 | 0.000000 | 0.000303 | 0.000000 | 0.000000 | 0.000000 | 0.000000 | 0.000615 | 0.000000 | 0.000000 | 0.000000 |
| 499 | 0.000000 | 0.000187 | 0.000000 | 0.000000 | 0.000000 | 0.000000 | 0.000379 | 0.000000 | 0.000000 | 0.000000 |
| 500 | 0.000000 | 0.000114 | 0.000000 | 0.000000 | 0.000000 | 0.000000 | 0.000232 | 0.000000 | 0.000000 | 0.000000 |
| 501 | 0.000000 | 0.000061 | 0.000000 | 0.000000 | 0.000000 | 0.000000 | 0.000125 | 0.000000 | 0.000000 | 0.000000 |
| 502 | 0.000000 | 0.000032 | 0.000000 | 0.000000 | 0.000000 | 0.000000 | 0.000064 | 0.000000 | 0.000000 | 0.000000 |
| 503 | 0.000000 | 0.000039 | 0.000000 | 0.000000 | 0.000000 | 0.000000 | 0.000079 | 0.000000 | 0.000000 | 0.000000 |
| 504 | 0.000000 | 0.000056 | 0.000000 | 0.000000 | 0.000000 | 0.000000 | 0.000113 | 0.000000 | 0.000000 | 0.000000 |
| 505 | 0.000000 | 0.000040 | 0.000000 | 0.000000 | 0.000000 | 0.000000 | 0.000082 | 0.000000 | 0.000000 | 0.000000 |
| 506 | 0.000000 | 0.000017 | 0.000000 | 0.000000 | 0.000000 | 0.000000 | 0.000034 | 0.000000 | 0.000000 | 0.000000 |
| 507 | 0.000000 | 0.000000 | 0.000000 | 0.000000 | 0.000000 | 0.000000 | 0.000000 | 0.000000 | 0.000000 | 0.000000 |
| 508 | 0.000000 | 0.000000 | 0.000000 | 0.000000 | 0.000000 | 0.000000 | 0.000000 | 0.000000 | 0.000000 | 0.000000 |
| 509 | 0.000000 | 0.000000 | 0.000000 | 0.000000 | 0.000000 | 0.000000 | 0.000000 | 0.000000 | 0.000000 | 0.000000 |
| 510 | 0.000000 | 0.000000 | 0.000000 | 0.000000 | 0.000000 | 0.000000 | 0.000000 | 0.000000 | 0.000000 | 0.000000 |
| 511 | 0.000000 | 0.000000 | 0.000000 | 0.000000 | 0.000000 | 0.000000 | 0.000000 | 0.000000 | 0.000000 | 0.000000 |
| 512 | 0.000000 | 0.000000 | 0.000000 | 0.000000 | 0.000000 | 0.000000 | 0.000000 | 0.000000 | 0.000000 | 0.000000 |
| 513 | 0.000000 | 0.000000 | 0.000000 | 0.000000 | 0.000000 | 0.000000 | 0.000000 | 0.000000 | 0.000000 | 0.000000 |
| 514 | 0.000000 | 0.000000 | 0.000000 | 0.000000 | 0.000000 | 0.000000 | 0.000000 | 0.000000 | 0.000000 | 0.000000 |
| 515 | 0.000000 | 0.000000 | 0.000000 | 0.000000 | 0.000000 | 0.000000 | 0.000000 | 0.000000 | 0.000000 | 0.000000 |
| 516 | 0.000000 | 0.000000 | 0.000080 | 0.000000 | 0.000000 | 0.000000 | 0.000000 | 0.000032 | 0.000000 | 0.000000 |
| 517 | 0.000000 | 0.000000 | 0.000109 | 0.000000 | 0.000000 | 0.000000 | 0.000000 | 0.000033 | 0.000000 | 0.000000 |
| 518 | 0.000000 | 0.000000 | 0.000120 | 0.000000 | 0.000000 | 0.000000 | 0.000000 | 0.000032 | 0.000000 | 0.000000 |
| 519 | 0.000000 | 0.000000 | 0.000115 | 0.000000 | 0.000000 | 0.000000 | 0.000000 | 0.000036 | 0.000000 | 0.000000 |
| 520 | 0.000000 | 0.000000 | 0.000146 | 0.000000 | 0.000000 | 0.000000 | 0.000000 | 0.000040 | 0.000000 | 0.000000 |
| 521 | 0.000000 | 0.000000 | 0.000182 | 0.000000 | 0.000000 | 0.000000 | 0.000000 | 0.000046 | 0.000000 | 0.000000 |
| 522 | 0.000000 | 0.000000 | 0.000209 | 0.000000 | 0.000000 | 0.000000 | 0.000000 | 0.000056 | 0.000000 | 0.000000 |
| 523 | 0.000000 | 0.000000 | 0.000273 | 0.000000 | 0.000000 | 0.000000 | 0.000000 | 0.000070 | 0.000000 | 0.000000 |
| 524 | 0.000000 | 0.000000 | 0.000352 | 0.000000 | 0.000000 | 0.000000 | 0.000000 | 0.000087 | 0.000000 | 0.000000 |
| 525 | 0.000000 | 0.000000 | 0.000431 | 0.000000 | 0.000000 | 0.000000 | 0.000000 | 0.000111 | 0.000000 | 0.000000 |
| 526 | 0.000000 | 0.000000 | 0.000547 | 0.000000 | 0.000000 | 0.000000 | 0.000000 | 0.000139 | 0.000000 | 0.000000 |
| 527 | 0.000000 | 0.000000 | 0.000605 | 0.000000 | 0.000000 | 0.000000 | 0.000000 | 0.000166 | 0.000000 | 0.000000 |
| 528 | 0.000000 | 0.000000 | 0.000705 | 0.000000 | 0.000000 | 0.000000 | 0.000000 | 0.000195 | 0.000000 | 0.000000 |
| 529 | 0.000000 | 0.000000 | 0.000833 | 0.000000 | 0.000000 | 0.000000 | 0.000000 | 0.000225 | 0.000000 | 0.000000 |
| 530 | 0.000000 | 0.000000 | 0.000995 | 0.000000 | 0.000000 | 0.000000 | 0.000000 | 0.000265 | 0.000000 | 0.000000 |
| 531 | 0.000000 | 0.000000 | 0.001231 | 0.000000 | 0.000000 | 0.000000 | 0.000000 | 0.000321 | 0.000000 | 0.000000 |
| 532 | 0.000000 | 0.000000 | 0.001584 | 0.000000 | 0.000000 | 0.000000 | 0.000000 | 0.000399 | 0.000000 | 0.000000 |
| 533 | 0.000000 | 0.000000 | 0.002145 | 0.000000 | 0.000000 | 0.000000 | 0.000000 | 0.000520 | 0.000000 | 0.000000 |
| 534 | 0.000000 | 0.000000 | 0.003084 | 0.000000 | 0.000000 | 0.000000 | 0.000000 | 0.000714 | 0.000000 | 0.000000 |
| 535 | 0.000000 | 0.000000 | 0.004446 | 0.000000 | 0.000000 | 0.000000 | 0.000000 | 0.001014 | 0.000000 | 0.000000 |
| 536 | 0.000000 | 0.000000 | 0.006337 | 0.000000 | 0.000000 | 0.000000 | 0.000000 | 0.001453 | 0.000000 | 0.000000 |
| 537 | 0.000000 | 0.000000 | 0.008447 | 0.000000 | 0.000000 | 0.000000 | 0.000000 | 0.002015 | 0.000000 | 0.000000 |
| 538 | 0.000000 | 0.000000 | 0.010130 | 0.000000 | 0.000000 | 0.000000 | 0.000000 | 0.002611 | 0.000000 | 0.000000 |
| 539 | 0.000000 | 0.000000 | 0.010810 | 0.000000 | 0.000000 | 0.000000 | 0.000000 | 0.003080 | 0.000000 | 0.000000 |
| 540 | 0.000000 | 0.000000 | 0.010670 | 0.000000 | 0.000000 | 0.000000 | 0.000000 | 0.003313 | 0.000000 | 0.000000 |
| 541 | 0.000000 | 0.000000 | 0.010040 | 0.000000 | 0.000000 | 0.000000 | 0.000000 | 0.003304 | 0.000000 | 0.000000 |
| 542 | 0.000000 | 0.000000 | 0.009381 | 0.000000 | 0.000000 | 0.000000 | 0.000000 | 0.003154 | 0.000000 | 0.000000 |
| 543 | 0.000000 | 0.000000 | 0.008592 | 0.000000 | 0.000000 | 0.000000 | 0.000000 | 0.002936 | 0.000000 | 0.000000 |
| 544 | 0.000000 | 0.000000 | 0.007566 | 0.000000 | 0.000000 | 0.000000 | 0.000000 | 0.002677 | 0.000000 | 0.000000 |
| 545 | 0.000000 | 0.000000 | 0.006217 | 0.000000 | 0.000000 | 0.000000 | 0.000000 | 0.002345 | 0.000000 | 0.000000 |
| 546 | 0.000000 | 0.000000 | 0.004532 | 0.000000 | 0.000000 | 0.000000 | 0.000000 | 0.001920 | 0.000000 | 0.000000 |
| 547 | 0.000000 | 0.000000 | 0.002791 | 0.000000 | 0.000000 | 0.000000 | 0.000000 | 0.001419 | 0.000000 | 0.000000 |
| 548 | 0.000000 | 0.000000 | 0.001545 | 0.000000 | 0.000000 | 0.000000 | 0.000000 | 0.000929 | 0.000000 | 0.000000 |
| 549 | 0.000000 | 0.000000 | 0.000904 | 0.000000 | 0.000000 | 0.000000 | 0.000000 | 0.000549 | 0.000000 | 0.000000 |
| 550 | 0.000000 | 0.000000 | 0.000510 | 0.000000 | 0.000000 | 0.000000 | 0.000000 | 0.000310 | 0.000000 | 0.000000 |
| 551 | 0.000000 | 0.000000 | 0.000248 | 0.000000 | 0.000000 | 0.000000 | 0.000000 | 0.000174 | 0.000000 | 0.000000 |
| 552 | 0.000000 | 0.000000 | 0.000152 | 0.000000 | 0.000000 | 0.000000 | 0.000000 | 0.000095 | 0.000000 | 0.000000 |
| 553 | 0.000000 | 0.000000 | 0.000085 | 0.000054 | 0.000000 | 0.000000 | 0.000000 | 0.000051 | 0.000000 | 0.000073 |
| 554 | 0.000000 | 0.000000 | 0.000044 | 0.000106 | 0.000000 | 0.000000 | 0.000000 | 0.000029 | 0.000000 | 0.000144 |
| 555 | 0.000000 | 0.000000 | 0.000000 | 0.000152 | 0.000000 | 0.000000 | 0.000000 | 0.000000 | 0.000000 | 0.000206 |
| 556 | 0.000000 | 0.000000 | 0.000000 | 0.000173 | 0.000000 | 0.000000 | 0.000000 | 0.000000 | 0.000000 | 0.000234 |
| 557 | 0.000000 | 0.000000 | 0.000000 | 0.000219 | 0.000000 | 0.000000 | 0.000000 | 0.000000 | 0.000000 | 0.000296 |
| 558 | 0.000000 | 0.000000 | 0.000000 | 0.000306 | 0.000000 | 0.000000 | 0.000000 | 0.000000 | 0.000000 | 0.000414 |
| 559 | 0.000000 | 0.000000 | 0.000000 | 0.000379 | 0.000000 | 0.000000 | 0.000000 | 0.000000 | 0.000000 | 0.000513 |
| 560 | 0.000000 | 0.000000 | 0.000000 | 0.000437 | 0.000000 | 0.000000 | 0.000000 | 0.000000 | 0.000000 | 0.000592 |
| 561 | 0.000000 | 0.000000 | 0.000000 | 0.000495 | 0.000000 | 0.000000 | 0.000000 | 0.000000 | 0.000000 | 0.000671 |
| 562 | 0.000000 | 0.000000 | 0.000000 | 0.000570 | 0.000000 | 0.000000 | 0.000000 | 0.000000 | 0.000000 | 0.000773 |
| 563 | 0.000000 | 0.000000 | 0.000000 | 0.000681 | 0.000000 | 0.000000 | 0.000000 | 0.000000 | 0.000000 | 0.000924 |
| 564 | 0.000000 | 0.000000 | 0.000000 | 0.000804 | 0.000000 | 0.000000 | 0.000000 | 0.000000 | 0.000000 | 0.001090 |
| 565 | 0.000000 | 0.000000 | 0.000000 | 0.000931 | 0.000000 | 0.000000 | 0.000000 | 0.000000 | 0.000000 | 0.001262 |
| 566 | 0.000000 | 0.000000 | 0.000000 | 0.001061 | 0.000000 | 0.000000 | 0.000000 | 0.000000 | 0.000000 | 0.001439 |
| 567 | 0.000000 | 0.000000 | 0.000000 | 0.001203 | 0.000000 | 0.000000 | 0.000000 | 0.000000 | 0.000000 | 0.001631 |
| 568 | 0.000000 | 0.000000 | 0.000000 | 0.001370 | 0.000000 | 0.000000 | 0.000000 | 0.000000 | 0.000000 | 0.001858 |
| 569 | 0.000000 | 0.000000 | 0.000000 | 0.001564 | 0.000000 | 0.000000 | 0.000000 | 0.000000 | 0.000000 | 0.002121 |
| 570 | 0.000000 | 0.000000 | 0.000000 | 0.001796 | 0.000000 | 0.000000 | 0.000000 | 0.000000 | 0.000000 | 0.002436 |
| 571 | 0.000000 | 0.000000 | 0.000000 | 0.002077 | 0.000000 | 0.000000 | 0.000000 | 0.000000 | 0.000000 | 0.002817 |
| 572 | 0.000000 | 0.000000 | 0.000000 | 0.002402 | 0.000000 | 0.000000 | 0.000000 | 0.000000 | 0.000000 | 0.003256 |
| 573 | 0.000000 | 0.000000 | 0.000000 | 0.002761 | 0.000000 | 0.000000 | 0.000000 | 0.000000 | 0.000000 | 0.003744 |
| 574 | 0.000000 | 0.000000 | 0.000000 | 0.003163 | 0.000000 | 0.000000 | 0.000000 | 0.000000 | 0.000000 | 0.004289 |
| 575 | 0.000000 | 0.000000 | 0.000000 | 0.003600 | 0.000000 | 0.000000 | 0.000000 | 0.000000 | 0.000000 | 0.004882 |
| 576 | 0.000000 | 0.000000 | 0.000000 | 0.004116 | 0.000000 | 0.000000 | 0.000000 | 0.000000 | 0.000000 | 0.005581 |
| 577 | 0.000000 | 0.000000 | 0.000000 | 0.004730 | 0.000000 | 0.000000 | 0.000000 | 0.000000 | 0.000000 | 0.006414 |
| 578 | 0.000000 | 0.000000 | 0.000000 | 0.005401 | 0.000000 | 0.000000 | 0.000000 | 0.000000 | 0.000000 | 0.007323 |
| 579 | 0.000000 | 0.000000 | 0.000000 | 0.006179 | 0.000000 | 0.000000 | 0.000000 | 0.000000 | 0.000000 | 0.008378 |
| 580 | 0.000000 | 0.000000 | 0.000000 | 0.007038 | 0.000000 | 0.000000 | 0.000000 | 0.000000 | 0.000000 | 0.009543 |
| 581 | 0.000000 | 0.000000 | 0.000000 | 0.008026 | 0.000000 | 0.000000 | 0.000000 | 0.000000 | 0.000000 | 0.010883 |
| 582 | 0.000000 | 0.000000 | 0.000000 | 0.009126 | 0.000000 | 0.000000 | 0.000000 | 0.000000 | 0.000000 | 0.012373 |
| 583 | 0.000000 | 0.000000 | 0.000000 | 0.010345 | 0.000000 | 0.000000 | 0.000000 | 0.000000 | 0.000000 | 0.014027 |
| 584 | 0.000000 | 0.000000 | 0.000000 | 0.011750 | 0.000000 | 0.000000 | 0.000000 | 0.000000 | 0.000000 | 0.015931 |
| 585 | 0.000000 | 0.000000 | 0.000000 | 0.013317 | 0.000000 | 0.000000 | 0.000000 | 0.000000 | 0.000000 | 0.018055 |
| 586 | 0.000000 | 0.000000 | 0.000000 | 0.015113 | 0.000000 | 0.000000 | 0.000000 | 0.000000 | 0.000000 | 0.020491 |
| 587 | 0.000000 | 0.000000 | 0.000000 | 0.017093 | 0.000000 | 0.000000 | 0.000000 | 0.000000 | 0.000000 | 0.023176 |
| 588 | 0.000000 | 0.000000 | 0.000000 | 0.019287 | 0.000054 | 0.000000 | 0.000000 | 0.000000 | 0.000018 | 0.026150 |
| 589 | 0.000000 | 0.000000 | 0.000000 | 0.021527 | 0.000128 | 0.000000 | 0.000000 | 0.000000 | 0.000042 | 0.029187 |
| 590 | 0.000000 | 0.000000 | 0.000000 | 0.023757 | 0.000178 | 0.000000 | 0.000000 | 0.000000 | 0.000058 | 0.032210 |
| 591 | 0.000000 | 0.000000 | 0.000000 | 0.025747 | 0.000161 | 0.000000 | 0.000000 | 0.000000 | 0.000052 | 0.034909 |
| 592 | 0.000000 | 0.000000 | 0.000000 | 0.027410 | 0.000143 | 0.000000 | 0.000000 | 0.000000 | 0.000046 | 0.037164 |
| 593 | 0.000000 | 0.000000 | 0.000000 | 0.028373 | 0.000196 | 0.000000 | 0.000000 | 0.000000 | 0.000064 | 0.038470 |
| 594 | 0.000000 | 0.000000 | 0.000000 | 0.028350 | 0.000272 | 0.000000 | 0.000000 | 0.000000 | 0.000088 | 0.038438 |
| 595 | 0.000000 | 0.000000 | 0.000000 | 0.027090 | 0.000338 | 0.000000 | 0.000000 | 0.000000 | 0.000110 | 0.036730 |
| 596 | 0.000000 | 0.000000 | 0.000000 | 0.024773 | 0.000353 | 0.000000 | 0.000000 | 0.000000 | 0.000115 | 0.033589 |
| 597 | 0.000000 | 0.000000 | 0.000000 | 0.021853 | 0.000387 | 0.000000 | 0.000000 | 0.000000 | 0.000126 | 0.029630 |
| 598 | 0.000000 | 0.000000 | 0.000000 | 0.018857 | 0.000415 | 0.000000 | 0.000000 | 0.000000 | 0.000135 | 0.025567 |
| 599 | 0.000000 | 0.000000 | 0.000000 | 0.016110 | 0.000463 | 0.000000 | 0.000000 | 0.000000 | 0.000151 | 0.021843 |
| 600 | 0.000000 | 0.000000 | 0.000000 | 0.013683 | 0.000518 | 0.000000 | 0.000000 | 0.000000 | 0.000169 | 0.018553 |
| 601 | 0.000000 | 0.000000 | 0.000000 | 0.011525 | 0.000572 | 0.000000 | 0.000000 | 0.000000 | 0.000186 | 0.015627 |
| 602 | 0.000000 | 0.000000 | 0.000000 | 0.009663 | 0.000637 | 0.000000 | 0.000000 | 0.000000 | 0.000208 | 0.013101 |
| 603 | 0.000000 | 0.000000 | 0.000000 | 0.008141 | 0.000740 | 0.000000 | 0.000000 | 0.000000 | 0.000241 | 0.011038 |
| 604 | 0.000000 | 0.000000 | 0.000000 | 0.006926 | 0.000888 | 0.000000 | 0.000000 | 0.000000 | 0.000289 | 0.009391 |
| 605 | 0.000000 | 0.000000 | 0.000000 | 0.005933 | 0.001048 | 0.000000 | 0.000000 | 0.000000 | 0.000341 | 0.008045 |
| 606 | 0.000000 | 0.000000 | 0.000000 | 0.005044 | 0.001188 | 0.000000 | 0.000000 | 0.000000 | 0.000387 | 0.006838 |
| 607 | 0.000000 | 0.000000 | 0.000000 | 0.004276 | 0.001318 | 0.000000 | 0.000000 | 0.000000 | 0.000429 | 0.005798 |
| 608 | 0.000000 | 0.000000 | 0.000000 | 0.003617 | 0.001477 | 0.000000 | 0.000000 | 0.000000 | 0.000481 | 0.004904 |
| 609 | 0.000000 | 0.000000 | 0.000000 | 0.003050 | 0.001619 | 0.000000 | 0.000000 | 0.000000 | 0.000527 | 0.004135 |
| 610 | 0.000000 | 0.000000 | 0.000000 | 0.002542 | 0.001810 | 0.000000 | 0.000000 | 0.000000 | 0.000590 | 0.003447 |
| 611 | 0.000000 | 0.000000 | 0.000000 | 0.002129 | 0.002029 | 0.000000 | 0.000000 | 0.000000 | 0.000661 | 0.002887 |
| 612 | 0.000000 | 0.000000 | 0.000000 | 0.001789 | 0.002294 | 0.000000 | 0.000000 | 0.000000 | 0.000747 | 0.002426 |
| 613 | 0.000000 | 0.000000 | 0.000000 | 0.001552 | 0.002556 | 0.000000 | 0.000000 | 0.000000 | 0.000833 | 0.002104 |
| 614 | 0.000000 | 0.000000 | 0.000000 | 0.001348 | 0.002838 | 0.000000 | 0.000000 | 0.000000 | 0.000925 | 0.001827 |
| 615 | 0.000000 | 0.000000 | 0.000000 | 0.001166 | 0.003142 | 0.000000 | 0.000000 | 0.000000 | 0.001023 | 0.001582 |
| 616 | 0.000000 | 0.000000 | 0.000000 | 0.001004 | 0.003507 | 0.000000 | 0.000000 | 0.000000 | 0.001142 | 0.001362 |
| 617 | 0.000000 | 0.000000 | 0.000000 | 0.000827 | 0.003971 | 0.000000 | 0.000000 | 0.000000 | 0.001293 | 0.001122 |
| 618 | 0.000000 | 0.000000 | 0.000000 | 0.000685 | 0.004554 | 0.000000 | 0.000000 | 0.000000 | 0.001484 | 0.000928 |
| 619 | 0.000000 | 0.000000 | 0.000000 | 0.000608 | 0.005176 | 0.000000 | 0.000000 | 0.000000 | 0.001686 | 0.000825 |
| 620 | 0.000000 | 0.000000 | 0.000000 | 0.000571 | 0.005781 | 0.000000 | 0.000000 | 0.000000 | 0.001883 | 0.000775 |
| 621 | 0.000000 | 0.000000 | 0.000000 | 0.000524 | 0.006421 | 0.000000 | 0.000000 | 0.000000 | 0.002092 | 0.000711 |
| 622 | 0.000000 | 0.000000 | 0.000000 | 0.000460 | 0.007163 | 0.000000 | 0.000000 | 0.000000 | 0.002333 | 0.000623 |
| 623 | 0.000000 | 0.000000 | 0.000000 | 0.000412 | 0.008062 | 0.000000 | 0.000000 | 0.000000 | 0.002626 | 0.000559 |
| 624 | 0.000000 | 0.000000 | 0.000000 | 0.000402 | 0.009093 | 0.000000 | 0.000000 | 0.000000 | 0.002962 | 0.000545 |
| 625 | 0.000000 | 0.000000 | 0.000000 | 0.000319 | 0.010198 | 0.000000 | 0.000000 | 0.000000 | 0.003322 | 0.000433 |
| 626 | 0.000000 | 0.000000 | 0.000000 | 0.000264 | 0.011320 | 0.000000 | 0.000000 | 0.000000 | 0.003687 | 0.000358 |
| 627 | 0.000000 | 0.000000 | 0.000000 | 0.000211 | 0.012510 | 0.000000 | 0.000000 | 0.000000 | 0.004075 | 0.000286 |
| 628 | 0.000000 | 0.000000 | 0.000000 | 0.000198 | 0.013737 | 0.000000 | 0.000000 | 0.000000 | 0.004475 | 0.000268 |
| 629 | 0.000000 | 0.000000 | 0.000000 | 0.000193 | 0.014960 | 0.000000 | 0.000000 | 0.000000 | 0.004873 | 0.000262 |
| 630 | 0.000000 | 0.000000 | 0.000000 | 0.000174 | 0.015940 | 0.000000 | 0.000000 | 0.000000 | 0.005192 | 0.000235 |
| 631 | 0.000000 | 0.000000 | 0.000000 | 0.000145 | 0.016623 | 0.000000 | 0.000000 | 0.000000 | 0.005415 | 0.000196 |
| 632 | 0.000000 | 0.000000 | 0.000000 | 0.000085 | 0.016903 | 0.000000 | 0.000000 | 0.000000 | 0.005506 | 0.000116 |
| 633 | 0.000000 | 0.000000 | 0.000000 | 0.000074 | 0.016627 | 0.000000 | 0.000000 | 0.000000 | 0.005416 | 0.000100 |
| 634 | 0.000000 | 0.000000 | 0.000000 | 0.000071 | 0.015650 | 0.000000 | 0.000000 | 0.000000 | 0.005098 | 0.000096 |
| 635 | 0.000000 | 0.000000 | 0.000000 | 0.000102 | 0.014097 | 0.000000 | 0.000000 | 0.000000 | 0.004592 | 0.000138 |
| 636 | 0.000000 | 0.000000 | 0.000000 | 0.000112 | 0.012247 | 0.000000 | 0.000000 | 0.000000 | 0.003989 | 0.000152 |
| 637 | 0.000000 | 0.000000 | 0.000000 | 0.000090 | 0.010467 | 0.000000 | 0.000000 | 0.000000 | 0.003410 | 0.000122 |
| 638 | 0.000000 | 0.000000 | 0.000000 | 0.000045 | 0.008839 | 0.000000 | 0.000000 | 0.000000 | 0.002879 | 0.000061 |
| 639 | 0.000000 | 0.000000 | 0.000000 | 0.000000 | 0.007473 | 0.000000 | 0.000000 | 0.000000 | 0.002434 | 0.000000 |
| 640 | 0.000000 | 0.000000 | 0.000000 | 0.000000 | 0.006298 | 0.000000 | 0.000000 | 0.000000 | 0.002052 | 0.000000 |
| 641 | 0.000000 | 0.000000 | 0.000000 | 0.000000 | 0.005306 | 0.000000 | 0.000000 | 0.000000 | 0.001728 | 0.000000 |
| 642 | 0.000000 | 0.000000 | 0.000000 | 0.000000 | 0.004477 | 0.000000 | 0.000000 | 0.000000 | 0.001458 | 0.000000 |
| 643 | 0.000000 | 0.000000 | 0.000000 | 0.000000 | 0.003809 | 0.000000 | 0.000000 | 0.000000 | 0.001241 | 0.000000 |
| 644 | 0.000000 | 0.000000 | 0.000000 | 0.000000 | 0.003303 | 0.000000 | 0.000000 | 0.000000 | 0.001076 | 0.000000 |
| 645 | 0.000000 | 0.000000 | 0.000000 | 0.000000 | 0.002915 | 0.000000 | 0.000000 | 0.000000 | 0.000949 | 0.000000 |
| 646 | 0.000000 | 0.000000 | 0.000000 | 0.000000 | 0.002583 | 0.000000 | 0.000000 | 0.000000 | 0.000841 | 0.000000 |
| 647 | 0.000000 | 0.000000 | 0.000000 | 0.000000 | 0.002244 | 0.000000 | 0.000000 | 0.000000 | 0.000731 | 0.000000 |
| 648 | 0.000000 | 0.000000 | 0.000000 | 0.000000 | 0.001920 | 0.000000 | 0.000000 | 0.000000 | 0.000625 | 0.000000 |
| 649 | 0.000000 | 0.000000 | 0.000000 | 0.000000 | 0.001654 | 0.000000 | 0.000000 | 0.000000 | 0.000539 | 0.000000 |
| 650 | 0.000000 | 0.000000 | 0.000000 | 0.000000 | 0.001460 | 0.000000 | 0.000000 | 0.000000 | 0.000475 | 0.000000 |
| 651 | 0.000000 | 0.000000 | 0.000000 | 0.000000 | 0.001239 | 0.000000 | 0.000000 | 0.000000 | 0.000404 | 0.000000 |
| 652 | 0.000000 | 0.000000 | 0.000000 | 0.000000 | 0.001006 | 0.000000 | 0.000000 | 0.000000 | 0.000328 | 0.000000 |
| 653 | 0.000000 | 0.000000 | 0.000000 | 0.000000 | 0.000753 | 0.000000 | 0.000000 | 0.000000 | 0.000245 | 0.000000 |
| 654 | 0.000000 | 0.000000 | 0.000000 | 0.000000 | 0.000601 | 0.000000 | 0.000000 | 0.000000 | 0.000196 | 0.000000 |
| 655 | 0.000000 | 0.000000 | 0.000000 | 0.000000 | 0.000527 | 0.000000 | 0.000000 | 0.000000 | 0.000172 | 0.000000 |
| 656 | 0.000000 | 0.000000 | 0.000000 | 0.000000 | 0.000462 | 0.000000 | 0.000000 | 0.000000 | 0.000150 | 0.000000 |
| 657 | 0.000000 | 0.000000 | 0.000000 | 0.000000 | 0.000370 | 0.000000 | 0.000000 | 0.000000 | 0.000120 | 0.000000 |
| 658 | 0.000000 | 0.000000 | 0.000000 | 0.000000 | 0.000280 | 0.000000 | 0.000000 | 0.000000 | 0.000091 | 0.000000 |
| 659 | 0.000000 | 0.000000 | 0.000000 | 0.000000 | 0.000243 | 0.000000 | 0.000000 | 0.000000 | 0.000079 | 0.000000 |
| 660 | 0.000000 | 0.000000 | 0.000000 | 0.000000 | 0.000229 | 0.000000 | 0.000000 | 0.000000 | 0.000075 | 0.000000 |
| 661 | 0.000000 | 0.000000 | 0.000000 | 0.000000 | 0.000215 | 0.000000 | 0.000000 | 0.000000 | 0.000070 | 0.000000 |
| 662 | 0.000000 | 0.000000 | 0.000000 | 0.000000 | 0.000197 | 0.000000 | 0.000000 | 0.000000 | 0.000064 | 0.000000 |
| 663 | 0.000000 | 0.000000 | 0.000000 | 0.000000 | 0.000188 | 0.000000 | 0.000000 | 0.000000 | 0.000061 | 0.000000 |
| 664 | 0.000000 | 0.000000 | 0.000000 | 0.000000 | 0.000175 | 0.000000 | 0.000000 | 0.000000 | 0.000057 | 0.000000 |
| 665 | 0.000000 | 0.000000 | 0.000000 | 0.000000 | 0.000199 | 0.000000 | 0.000000 | 0.000000 | 0.000065 | 0.000000 |
| 666 | 0.000000 | 0.000000 | 0.000000 | 0.000000 | 0.000195 | 0.000000 | 0.000000 | 0.000000 | 0.000064 | 0.000000 |
| 667 | 0.000000 | 0.000000 | 0.000000 | 0.000000 | 0.000170 | 0.000000 | 0.000000 | 0.000000 | 0.000055 | 0.000000 |
| 668 | 0.000000 | 0.000000 | 0.000000 | 0.000000 | 0.000132 | 0.000000 | 0.000000 | 0.000000 | 0.000043 | 0.000000 |
| 669 | 0.000000 | 0.000000 | 0.000000 | 0.000000 | 0.000116 | 0.000000 | 0.000000 | 0.000000 | 0.000038 | 0.000000 |
| 670 | 0.000000 | 0.000000 | 0.000000 | 0.000000 | 0.000084 | 0.000000 | 0.000000 | 0.000000 | 0.000027 | 0.000000 |
| 671 | 0.000000 | 0.000000 | 0.000000 | 0.000000 | 0.000061 | 0.000000 | 0.000000 | 0.000000 | 0.000020 | 0.000000 |
| 672 | 0.000000 | 0.000000 | 0.000000 | 0.000000 | 0.000041 | 0.000000 | 0.000000 | 0.000000 | 0.000013 | 0.000000 |
| 673 | 0.000000 | 0.000000 | 0.000000 | 0.000000 | 0.000048 | 0.000000 | 0.000000 | 0.000000 | 0.000016 | 0.000000 |
| 674 | 0.000000 | 0.000000 | 0.000000 | 0.000000 | 0.000077 | 0.000000 | 0.000000 | 0.000000 | 0.000025 | 0.000000 |
| 675 | 0.000000 | 0.000000 | 0.000000 | 0.000000 | 0.000099 | 0.000000 | 0.000000 | 0.000000 | 0.000032 | 0.000000 |
| 676 | 0.000000 | 0.000000 | 0.000000 | 0.000000 | 0.000090 | 0.000000 | 0.000000 | 0.000000 | 0.000029 | 0.000000 |
| 677 | 0.000000 | 0.000000 | 0.000000 | 0.000000 | 0.000041 | 0.000000 | 0.000000 | 0.000000 | 0.000013 | 0.000000 |
| 678 | 0.000000 | 0.000000 | 0.000000 | 0.000000 | 0.000000 | 0.000000 | 0.000000 | 0.000000 | 0.000000 | 0.000000 |
| 679 | 0.000000 | 0.000000 | 0.000000 | 0.000000 | 0.000000 | 0.000000 | 0.000000 | 0.000000 | 0.000000 | 0.000000 |
| 680 | 0.000000 | 0.000000 | 0.000000 | 0.000000 | 0.000000 | 0.000000 | 0.000000 | 0.000000 | 0.000000 | 0.000000 |
| 681 | 0.000000 | 0.000000 | 0.000000 | 0.000000 | 0.000000 | 0.000000 | 0.000000 | 0.000000 | 0.000000 | 0.000000 |
| 682 | 0.000000 | 0.000000 | 0.000000 | 0.000000 | 0.000000 | 0.000000 | 0.000000 | 0.000000 | 0.000000 | 0.000000 |
| 683 | 0.000000 | 0.000000 | 0.000000 | 0.000000 | 0.000000 | 0.000000 | 0.000000 | 0.000000 | 0.000000 | 0.000000 |
| 684 | 0.000000 | 0.000000 | 0.000000 | 0.000000 | 0.000000 | 0.000000 | 0.000000 | 0.000000 | 0.000000 | 0.000000 |
| 685 | 0.000000 | 0.000000 | 0.000000 | 0.000000 | 0.000000 | 0.000000 | 0.000000 | 0.000000 | 0.000000 | 0.000000 |
| 686 | 0.000000 | 0.000000 | 0.000000 | 0.000000 | 0.000000 | 0.000000 | 0.000000 | 0.000000 | 0.000000 | 0.000000 |
| 687 | 0.000000 | 0.000000 | 0.000000 | 0.000000 | 0.000000 | 0.000000 | 0.000000 | 0.000000 | 0.000000 | 0.000000 |
| 688 | 0.000000 | 0.000000 | 0.000000 | 0.000000 | 0.000000 | 0.000000 | 0.000000 | 0.000000 | 0.000000 | 0.000000 |
| 689 | 0.000000 | 0.000000 | 0.000000 | 0.000000 | 0.000000 | 0.000000 | 0.000000 | 0.000000 | 0.000000 | 0.000000 |
| 690 | 0.000000 | 0.000000 | 0.000000 | 0.000000 | 0.000000 | 0.000000 | 0.000000 | 0.000000 | 0.000000 | 0.000000 |
| 691 | 0.000000 | 0.000000 | 0.000000 | 0.000000 | 0.000000 | 0.000000 | 0.000000 | 0.000000 | 0.000000 | 0.000000 |
| 692 | 0.000000 | 0.000000 | 0.000000 | 0.000000 | 0.000000 | 0.000000 | 0.000000 | 0.000000 | 0.000000 | 0.000000 |
| 693 | 0.000000 | 0.000000 | 0.000000 | 0.000000 | 0.000000 | 0.000000 | 0.000000 | 0.000000 | 0.000000 | 0.000000 |
| 694 | 0.000000 | 0.000000 | 0.000000 | 0.000000 | 0.000000 | 0.000000 | 0.000000 | 0.000000 | 0.000000 | 0.000000 |
| 695 | 0.000000 | 0.000000 | 0.000000 | 0.000000 | 0.000000 | 0.000000 | 0.000000 | 0.000000 | 0.000000 | 0.000000 |
| 696 | 0.000000 | 0.000000 | 0.000000 | 0.000000 | 0.000000 | 0.000000 | 0.000000 | 0.000000 | 0.000000 | 0.000000 |
| 697 | 0.000000 | 0.000000 | 0.000000 | 0.000000 | 0.000000 | 0.000000 | 0.000000 | 0.000000 | 0.000000 | 0.000000 |
| 698 | 0.000000 | 0.000000 | 0.000000 | 0.000000 | 0.000000 | 0.000000 | 0.000000 | 0.000000 | 0.000000 | 0.000000 |
| 699 | 0.000000 | 0.000000 | 0.000000 | 0.000000 | 0.000000 | 0.000000 | 0.000000 | 0.000000 | 0.000000 | 0.000000 |
| 700 | 0.000000 | 0.000000 | 0.000000 | 0.000000 | 0.000000 | 0.000000 | 0.000000 | 0.000000 | 0.000000 | 0.000000 |
